# Study on the Deformation Measurement of the Cast-In-Place Large-Diameter Pile Using Fiber Bragg Grating Sensors

**DOI:** 10.3390/s17030505

**Published:** 2017-03-03

**Authors:** Lei Gao, Kai Yang, Xiaorui Chen, Xiangjuan Yu

**Affiliations:** 1 Key Laboratory of Ministry of Education for Geomechanics and Embankment Engineering, Hohai University, Nanjing 210098, China; yangkwasaima@163.com (K.Y.); cxr245711247@163.com (X.C.); xjyu@hhu.edu.cn (X.Y.); 2 Research Institute of Geotechnical Engineering, Hohai University, Nanjing 210098, China

**Keywords:** PCC pile, strain, deformation, fiber Bragg grating (FBG), sensor

## Abstract

Compared with conventional piles such as the circle pile, the cast-in-place large-diameter pile (PCC pile) has many advantages: the lateral area of PCC pile is larger and the bearing capacity of PCC pile is higher. It is more cost-effective than other piles such as square pile under the same condition. The deformation of the PCC pile is very important for its application. In order to obtain the deformation of the PCC pile, a new type of quasi-distributed optical fiber sensing technology named a fiber Bragg grating (FBG) is used to monitor the deformation of the PCC pile. The PCC model pile is made, the packaging process of the PCC model pile and the layout of fiber sensors are designed, and the strains of the PCC model pile based on FBG sensors are monitored. The strain of the PCC pile is analyzed by the static load test. The results show that FBG technology is successfully applied for monitoring the deformation of the PCC pile, the monitoring data is more useful for the PCC pile. It will provide a reference for the engineering applications.

## 1. Introduction

As a new soft foundation reinforcement technology, the cast-in-place large-diameter pile (PCC pile) has many advantages. With the large diameter of the PCC pile, the amount of concrete is little and the bearing capacity of pile is higher. Compared with other solid concrete piles, the PCC pile has a large contact area between the pipe pile and the soil mass, and it increases the friction resistance of the pile. At the same time, the diameter of the PCC pile is more than 1 m, the per unit area using pile is less under the same load, it can effectively reduce the amount of work and save expenditure. The PCC pile can be used in the highway, high-speed railway, municipal roads, and other large area soft foundation treatment engineering projects, and it achieves good social and economic benefits. Liu Hanlong and others carried out a series of PCC pile designs, engineering applications, and testing, and it was put forward that the pile has high bearing capacity, high quality, low cost, and so on. The large scale model test was used to analyze the pile soil interaction characteristics and bearing capacity of the PCC pile under horizontal load. The variable impedance of the computational model and wave equation of the PCC pile are studied [1,2,3,4,5].

Recently, the optical fiber sensing technology has been used in the monitoring of civil engineering structures [5,6,7,8,9,10,11]. Fiber Bragg gratings (FBG), as one of the optical fiber technologies, have many advantages such as the distributed, interference, and corrosion resistance. Compared to the conventional monitoring technologies, FBG technology is formed by using the phase mask method to change the refractive index of the optical fiber [12,13,14,15]. The FBG sensors can measure the distribution of strain, temperature, displacements, and so on with high accuracy and stability [16,17,18,19,20,21,22,23]. This technology has been successfully applied to monitor the field performance of reinforced slopes, dikes, and bridges.

The deformation characteristics of the PCC pile are very important for the optimum design and construction of the PCC pile. However, as a new type of pile, the strain distribution, bearing characteristics, and other aspects of the PCC pile are still not clear for its application. At present, the traditional strain gauges are used to monitor the strain of the pile body, but the traditional strain sensor is easy to destroy and it has electromagnetic interference, the full deformation characteristics of the PCC pile cannot be obtained, and it is difficult to achieve the optimal design and construction of the PCC pile. Therefore, it is important to study the deformation characteristics of the PCC pile by using a new technology and method, which is important for the application of the PCC pile. FBG as the latest optical monitoring technology have high monitoring precision of structures and concrete. 

The new optical fiber sensing technology (FBG) will be used to monitor the deformation of the PCC pile, the special packaging of the optical fiber sensor, the coordination of the pile body, the protection technology of the optical fiber sensor, and the data analysis and processing of the optical fiber monitoring technology, all of which are studied in this paper. Moreover, the deformation characteristics of the PCC pile are analyzed. The strain distribution of the PCC pile is obtained to provide basic data, which will provide a new understanding of the PCC pile through the static load model test. This study will provide technical support for the optimization design, construction, and application for the PCC pile.

## 2. Materials and Methods

### 2.1. The PCC Model Pile and Soil

In order to obtain the deformation of the PCC pile, it is important to learn whether the bearing capacity of the PCC pile can meet the design requirement, so it is necessary to carry out the static load test for the PCC pile. There are two main experimental methods: one is the model test, and the other is the field test. Through a certain test method and monitoring method, the characteristics and laws of the PCC pile can be directly observed. For a more in-depth understanding, it can grasp the law of the PCC pile to provide a good foundation. The deformation of the pile foundation under the action of the load is difficult to obtain, and the stress state is very complex. The field static load test needs to take into account the instrument layout, the surrounding environment conditions, the loading conditions, and a series of complex problems. As such, the results cannot reflect the deformation of the pile body. The field test is not repeatable when the test data is not ideal and will waste manpower and material resources. In light of the above problems, the model test has become an indispensable part for stress and strain variations of the pile foundation in the study. Considering the application of FBG sensors to the PCC pile for the first time, in order to obtain a good strain curve of the pile body, the model test is chosen because it can accurately control the experimental condition only in this way and can avoid adverse effects of the environmental conditions of the site. Therefore, a correct analysis is needed for the PCC pile under the vertical static loading. The purpose of this experiment is mainly so that FBG technology can be applied to the monitoring of the strain of a PCC pile. 

**Figure 1:**
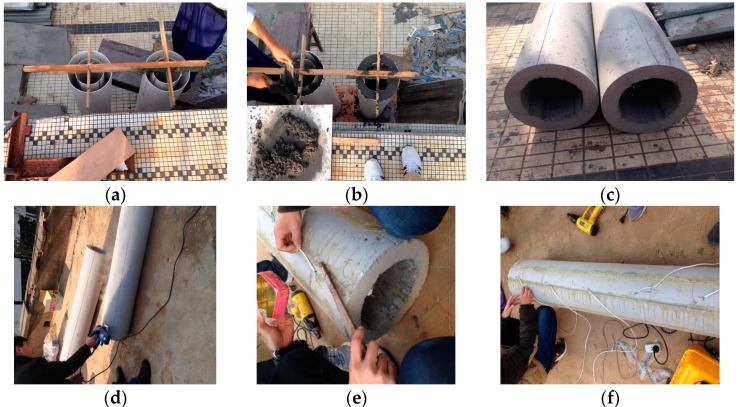
Process of fiber Bragg grating (FBG) sensor package. (**a**) Fixed the moulds; (**b**) Concreting; (**c**) the PCC model pile; (**d**) Fixed and Cut; (**e**) Fixed-point paste; (**f**) Comprehensive paste.

The processes of the model pile for the PCC pile are customized mainly to make a model pile mold, a concrete mixture ratio is determined, the pouring pile is maintenance, and the maintenance is removed after forming. It can be seen in Figure 1a–c. The common specification of the PCC pile is an outer diameter of 1000 mm and a wall thickness of 120 mm. Based on this situation, the model pile for the PCC pile is designed for an outer diameter of 315 mm and an internal diameter of 200 mm. In order to ensure that the cast is in place to meet the design requirements of the PCC pile, the concrete mix ratio is chosen. The weight ratio of cement/water/sand/gravel is 1:0.47:1.59:3.39. The parameters of the PCC model pile are shown in Table 1. 

According to the design of the section size of the PCC pile, the PCC model pile is designed. Because the PCC pile is a new type of pile, it is difficult to build a large diameter pipe pile model. Taking into account the cost and construction process of the PCC pile, the design of the PCC pile material used the circular PVC plastic pipe, and two types of PVC pipes are obtained for the diameter of 200 mm and 315 mm, respectively. In order to analyze the strain difference between the inner and outer wall of the PCC pile body, it is necessary to keep the inner wall of the PCC pile in advance to lay the optical fiber sensor, and it will have the purpose of monitoring the inner deformation of the PCC pile. The inner wall of the 200 mm PVC pipe is drilled, and the 2 mm of fine steel wire is then fixed to the pipe so that the groove is built in the concrete when the concrete is poured into the PVC pipe. When pouring concrete, the pipe ring is small. The concrete should be poured into the ring with a small shovel. 

In the process of laying FBG sensors, the coordination of deformation and the coordination of FBG sensors with the PCC pile are needed to consider the survival rate of FBG sensors, and the survival rate should be enough during the test. The mobile, embedded loading will inevitably produce friction, collision, or other damage on the PCC pile. If the FBG sensors are directly exposed to the surface of the PCC model pile, they are very easy to break, and it is not guaranteed that the FBG sensors along the direction of the axial force of the pile are still in good condition. Therefore, it needs to slot along the pile body, and the depth of the slot is about 2~3 mm. The optical fiber sensor can be wrapped completely in the groove of the pile body, and the friction is not affected by the optical fiber. The slot should be kept in the direction of the groove along the pile so that bending is avoided. At the same time, the groove should be smooth to prevent slotting too deep or shallow, and the out-of-flatness of groove will lead the optical fiber bending to affect the monitoring data quality on the inside. Because the FBG sensors are only monitoring the axial strain in the PCC pile, at the beginning, the process of the fixed line slot has a great influence on the monitoring data. The direction of the slot and the monitoring data can correctly reflect the strain characteristics of the PCC pile body. The sensing fiber has a flexible structure, the model pile has a rigid structure, and, in order to accurately obtain the deformation of the PCC model pile, the optical fiber must be closely connected with the model pile. The deformation and coordination can thus be achieved. Therefore, the packaging, the selection of the binder, the layout, and other aspects of the process should meet this requirement. The binder and two kinds of epoxy resin AB glue are used. The sensing optical fiber is thin and fragile, especially on the outside of the PCC pile; the layout process and test process is easy to damage, and the lower survival rate of the sensors will affect the collection of the test data. The protection of the sensing optical fiber is an important step in the test. Therefore, the optical fiber can be exposed on the outside of the hose connection protection in the layout and packaging process. Then, a soft cotton is used to protect the optical fiber, which will prevent possible damage during the preparation of the test. The specific layout steps are as follows and can be seen in Figure 1d–f.

- The line slot and point are fixed along the direction of the PCC pile with the ring. The worker set aside in the groove corresponding to 2 mm of the groove and cut out about 2 mm for the groove. In a PCC pile body under the selection of symmetrical grooves, the paste line of FBG sensors is fixed. On the selected groove, with a marker of the FBG points from the pile top with 20 cm, the middle is every 30 cm between two raster points, with a total of 5 points.
- An optical fiber loose tube is used at the top of the pile, which enables the 0.9 mm optical fiber in the optical fiber loose tube to strengthen the protection, and this will avoid the destruction.
- For the fixed-point paste, the FBG sensor is fixed in the predetermined layout position; the AB glue is used for the FBG sensors around the paste and fixed in the groove of the PCC pile, which provides the FBG sensors with very little pre-stress.
- For the protecting, the epoxy resin adhesive is used and fixed in the groove to protect the FBG sensors.

Due to the dual sensitivity of the temperature and strain of the sensing fiber, the influence of temperature variation on the strain of the PCC model pile is considered in the test process. In the case of the sensing optical fiber is not affected by the force, the strain of optical fiber is caused by temperature of the surrounding soil. Therefore, the influence of temperature on the strain of the PCC pile should be monitored in the test process. In order to obtain the real strain of the PCC model pile under the vertical load, the effect of the temperature on the strain of the pile is considered, and a hollow PVC tube is buried near the PCC pile with a spacing of 30 cm. In the monitoring of the pile deformation process, the FBG sensors are fixed into the tube, sand is added so that it can monitor the temperature of soil. In this case, the temperature of the soil can be obtained, it can be used to eliminate the temperature effect on the strain of the PCC pile, and the deformation characteristic of the PCC model pile in the static load is obtained. 

In order to simulate the situation, the soft soil foundation is simplified, the clay soil around the pile has a height of 0.6 m, and the lower layer uses sand with a height of 1.6 m. Soil samples are taken from the model and the soil test is carried out, and the physical properties of the soil layer are obtained as shown in Table 2.

### 2.2. Static Load Model Test of the PCC Pile

**Figure 2:**
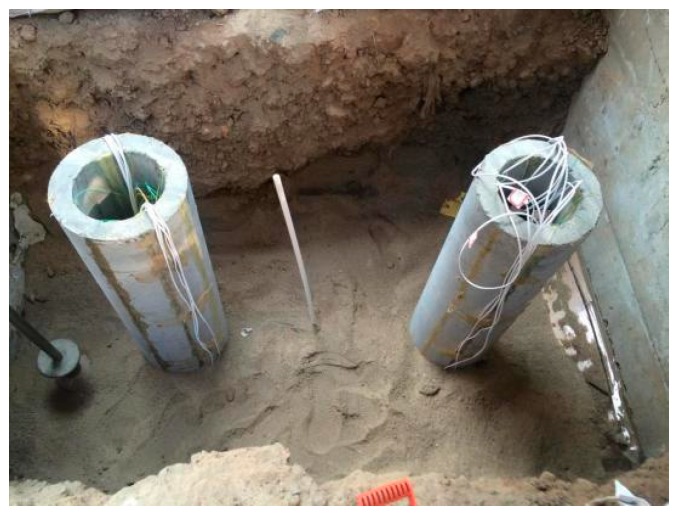
Soil layered compaction.

In order to ensure that the soil can accurately simulate the performance of the actual soil, the soil is compacted at 20 cm. It is careful that the hammer does not destroy the optical fiber sensor on the pile when the soil is compacted. It is set aside for two weeks to ensure that the clay soil can be consolidated, as shown in Figure 2.

**Figure 3:**
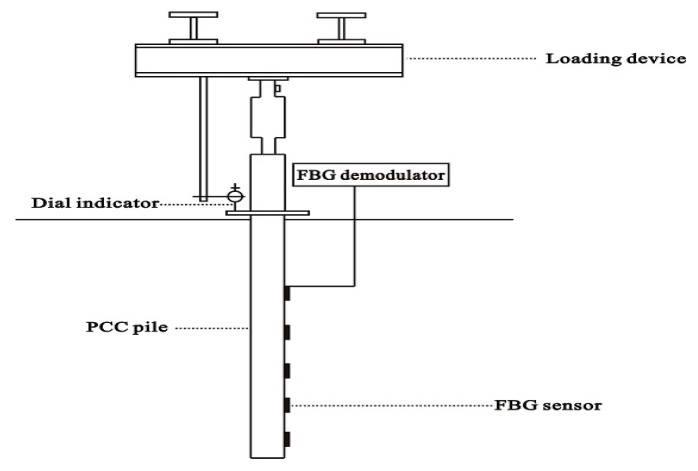
Static load model test.

Figure 3 shows the configuration of the static load model test. The loading system is mainly composed of loading equipment with digital display equipment, the anti-force frame, and a pressure transmission device. In order to ensure the loading process, the digital equipment is specially equipped. The magnitude of load and the change of load can be reflected in a timely manner by the digital display equipment. The model test equipment uses the reinforced concrete structure, the length of model is 265 cm, the width is 200 cm, and the height is 230 cm. The model trough is equipped with stairs, and the organic glass window is used to observe the filling and stress of soil after loading. The channel on both sides of the main girder end with a model groove, and the main beam and secondary beam is connected. Because the counter force frame is through the channel and is with the model, the load by the channel steel model groove transfers to the model at the bottom of the trench so that it guarantees the counter force frame for the security of the load transfer. The counter force frame consists of two main beams and secondary beams made of special steel. Two main beams are 150 cm, and the overall span of the main beam is 235 cm; the secondary beam is 100 cm, and the overall span of the secondary beam is 150 cm. The pressure equipment uses the Jack, the height of the Jack is 300 mm, the diameter of the piston is 80 mm, the maximum stroke is 200 mm, and the maximum load is 250 kN. The static load test uses FBG technology as well as the traditional resistance strain gauge, and the strain of inner wall and outer wall of the pile are monitored. The FBG sensor used in this experiment is produced by Suzhou Nanzhi Company of China. The wavelength range of FBG Sensors is 1510~1590 nm. The FBG demodulator is a SM125 demodulator produced by the Optics Micron Company, (852 Century Place NE, Atlanta, GA 30345 America) and the SM125 demodulator is designed to measure the stress, temperature, and pressure of the low speed variation. The scanning frequency of each channel with 1 Hz can be monitored at the same time. While SM125 can be extended to 16 channels at any time to monitor more than 40 FBG sensors, SM125 is mainly based on the demodulation technology of the fiber Fabry Perot filter and is suitable for long-term monitoring. The resolution of SM125 demodulator is 2.5 pm.

The FBG sensors are made of a variety of sensors such as temperature, strain, stress, acceleration, and pressure sensors. Different FBG sensors can have different working wavelengths, so they can use wave division multiplexing technology in a single optical fiber cascaded multiple FBG sensor for distributed measurement. It has a small size, a light weight, compatibility with optical fibers, low insertion loss, good long-term stability, and so on. It is particularly suitable for use in harsh environments that are inflammable, explosive, or substantially electromagnetic. When the temperature or stress changes, the fiber produces an axial strain, which makes the grating period change; at the same time, the refractive index of the optical fiber changes, which causes a wavelength shift in the grating. By using the linear relationship between the strain and wavelength shift of the grating, the deformation of the measured object can be calculated. When the strain of the pile is changed, the optical fiber grating wavelength change of the pile surface is monitored, and it can obtain the strain of the PCC pile under the vertical load. When the FBG wavelength changed with 0.001 nm, the corresponding strain value is 1 με. 

The maximum load is 40 kN in this test, which is performed 4 times, and the increment of vertical load at each level is 10 kN. The load will gradually increase and reach stability. After the completion of the loading, a dial indicator data is read every two hours; when the per hour settlement is less than 0.1 mm, the pile is in a steady state under the load operation. Under the condition of a constant vertical load, the vertical displacement increases rapidly, the speed increases gradually, when the maximum load or displacement is reached, it must stop the experiment. When the pile is crushed, the loading should be stopped, thus concluding the test.

## 3. Results

**Figure 4:**
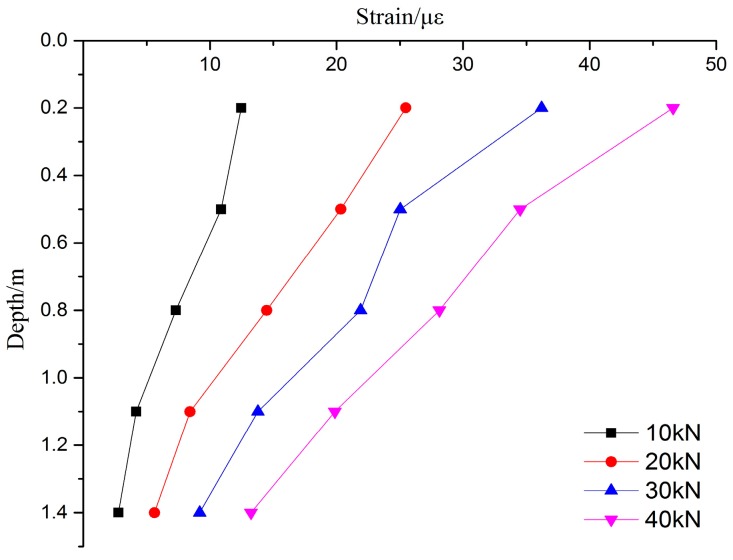
Strain curve of outer wall for the PCC model pile.

**Figure 5:**
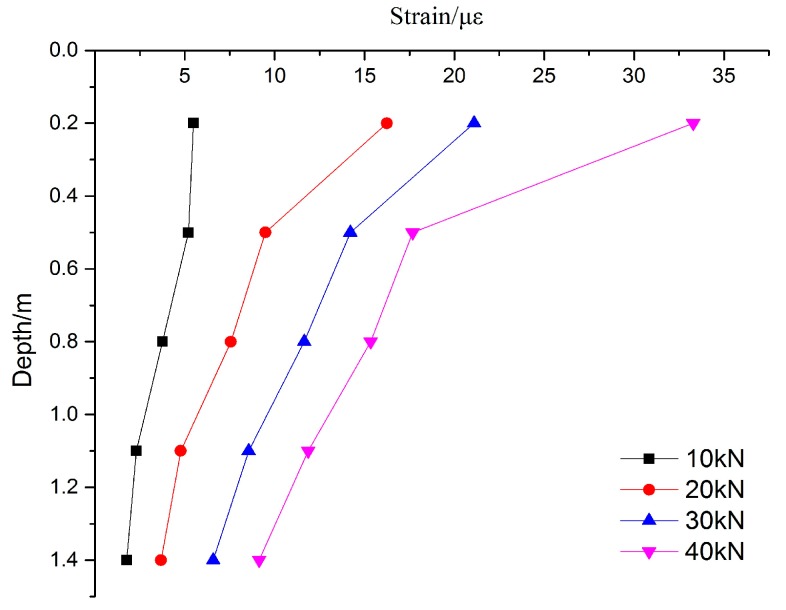
Strain curve of the inner wall for the PCC model pile.

The strain of the PCC pile is monitored by FBG technology, the FBG sensors are used to monitor the deformation of the PCC pile, and the FBG sensors are arranged on the inner and outer wall of the PCC pile body. The deformation characteristics of the PCC pile can thus be more accurately analyzed. The FBG sensing fibers are used to connect the four lines of the FBG fiber sensors together. the model test is small, and the pile body is shorter with only 1.5 m. In order to ensure the survival rate of the optical fiber sensors, the single end of the layout is easy to slot and layout, and it can reduce the slotted pile body and the coated epoxy resin. This can meet the requirements of the pile body and fiber sensor. In the process of monitoring based on FBG, the effect of temperature compensation of strain on the pile is eliminated. After the completion of each loading, the FBG wavelengths are read, and the groups of wavelength change chart are obtained. Four sets of data with 10 kN, 20 kN, 30 kN, and 40 kN are selected for comparison and analysis. The results of the FBG method of the PCC pile are shown in Figure 4 and Figure 5, and the variation of strain and deformation characteristics for the PCC model pile can be seen.

On the whole, the inner and outer wall deformation of the PCC pile body are consistent with the overall change in the law, the strain in the upper part of the decline rate is fast, and the lower part of the rate decreases in speed. It can be seen that, when the loading increases, the strain of the PCC pile also increases. The strain of the PCC pile decreases with depth under the same loading. This indirectly proves the reliability of FBG monitoring for the strain of the PCC pile body. The strain of the inner wall is much smaller than that of the outer wall. This may be due to the small model pile used in this experiment. Within a diameter of only 20 cm, there is less pile core soil, and it is difficult to form the same surrounding environment with the outer pile body, so the strain of the inner wall for the PCC pile is small. In order to study the stress and strain variation law of the pile body, the mean value method will be applied to calculate the stress on the inner and the outer wall. According to the section size of the model pile, the elastic modulus and the pile strain can be used to calculate the axial force of the PCC pile. The declining rate of the inner and outer wall strain of the PCC pile is higher in the upper part and smaller in the lower. This shows that the upper part of the clay is provided with a side frictional resistance greater than that provided by the lower sand. The upper layer of the soil is clay, the thickness of the soil is 0.6 m, and the lower soil is sand, which indirectly shows that the soil around the pile body has different effects on the mechanical properties of the PCC pile. As shown in Figure 4 and Figure 5, the distribution of strains of the PCC model pile under static load at all levels is slowly decreasing. With the increase of the load, the bearing capacity of the PCC pile gradually increases. The strain monitoring data based on the FBG is consistent, indicating that the application of FBG technology for the PCC pile is feasible for monitoring the strain. The model test is very successful, the method and process of the PCC pile based on the FBG are reliable, and the strain data of the FBG sensors are very useful for the PCC pile. It is worth noting that when the load is applied, there is a certain degree of separation between the pile and the counter force frame, because the soil under the bottom of pile is compressed and the pile is sinking, so the top of the pile force cannot accurately reach the set value of the load.

## 4. Conclusions

In this paper, a new optical fiber monitoring technology named FBG is used to monitor the deformation of the PCC pile in combination with the static load model test. Based on the strain data of the PCC pile, the deformation characteristics of the PCC pile were studied.

(1) Combined with the PCC pile, the construction of the PCC model pile in the static load test was developed; the FBG sensors were first used in the PCC pile, and the fiber laying and protection process were introduced in detail.
(2) According to the monitoring strain data of the PCC pile body based on FBG sensors, the strain curve distribution of the PCC pile body were obtained under a different loading, and the deformation variation characteristics of the PCC pile were studied.
(3) The inner wall and outer wall deformation of the pile body is consistent with the overall change in the law; the strain in the upper part of the decline rate is fast, and the lower part of the rate decreases in speed. It can be seen that, when the loading increases, the strain of the PCC pile also increases. The strain of the PCC pile decreases with the depth under the same loading. This indirectly proves the reliability of the FBG technology for the strain of the PCC pile body. The strain on the inner wall is much smaller than that of the outer wall, because the model pile used in this experiment is small and there is less soil in the inner wall of the pile than there is in the outer wall of the pile.

This study provides scientific information support and technical support for the optimization design, construction, and application of PCC piles.

## Figures and Tables

**Table 1:** Parameters of the cast-in-place large-diameter (PCC) model pile.

Parameter	Unit	Model Pile	Parameter	Unit	Model Pile
Area	*S* (m^2^)	0.046	Pile length	*L* (m)	1.5
Circumference	*C* (m)	0.989	Internal diameter	*d* (m)	0.2
External diameter	*D* (m)	0.315	Volume	*V* (m^3^)	0.070

**Table 2:** Physical indexes of soil layers.

Material	Soil Water Content *w* (%)	Natural Density *ρ* (g/cm^3^)	Thickness (m)
clay	19.22	1.93	0.6
sand	1.33	1.70	1.6
